# Prevalence of Methicillin Resistant Staphylococcus aureus Nasal Colonizers among Basic Science MBBS and BDS Students of Kathmandu Medical College

**DOI:** 10.31729/jnma.5561

**Published:** 2021-01-31

**Authors:** Manisha Sharma, Beena Jha, Chandra Prakash Bhatt

**Affiliations:** 1 Department of Microbiology, Kathmandu Medical College and Teaching Hospital, Sinamangal, Kathmandu, Nepal

**Keywords:** *antibiotic resistance*, *MRSA*, *nasal colonization*, *Staphylococcus aureus*

## Abstract

**Introduction:**

Methicillin-Resistant Staphylococcus aureus exhibit multiple drug resistance phenotypes. Colonizers harboring Methicillin-Resistant Staphylococcus aureus are often associated with its outbreaks in both hospital and community settings. This study was done to determine the prevalence of nasal carriage rate of Methicillin-Resistant Staphylococcus aureus among basic science MBBS and BDS students of Kathmandu Medical College.

**Methods:**

A descriptive cross-sectional study was done in Kathmandu Medical College from March 5 to June 5 2020. Ethical clearance was obtained from the Institutional Review Committee with reference no. 040320201. A convenient sampling method was used, and the sample size was calculated with a prevalence of 50%. Two hundred students studying MBBS and BDS were enrolled.

The nasal swab was collected and processed using standard microbiological methods. The data obtained were computed and analyzed using Statistical Package for Social Sciences 16.0 Version.

**Results:**

Among 200 participants, 9 (4.5%) were found to be nasal carriers of Methicillin-Resistant Staphylococcus aureus.

**Conclusions:**

Colonization of anterior nares by Methicillin-Resistant Staphylococcus aureus in apparently healthy individuals is a cause of concern. Education regarding Methicillin-Resistant Staphylococcus aureus, its carrier and significance, and its screening must be included early on in MBBS and BDS.

## INTRODUCTION

Methicillin-resistant Staphylococcus aureus (MRSA), once confined mainly to healthcare-associated infections, has been increasingly reported from community.^1^ The hospital-associated MRSA (HA-MRSA) is generally associated with patients with predisposing factors such as prolonged hospitalization, use of indwelling catheters, or prior surgical procedures. In contrast, community-associated MRSA (CA-MRSA) is associated with healthy and younger people without such predisposing factors.^2^ It has been identified that nasal colonization of S. aureus plays a key role in its pathogenesis.^3^

MRSA isolates exhibit multidrug resistance; moreover, increasing reports of MRSA isolates with decreased susceptibility to glycopeptides (glycopeptide intermediately susceptible S. aureus, GISA) is a cause for great public concern.^4^

In this study, we aimed to ascertain the prevalence of nasal colonization rate of MRSA among healthy basic science MBBS and BDS students of Kathmandu Medical College.

## METHODS

A descriptive cross-sectional study was done in Kathmandu Medical College from 5^th^ March to 5^th^ June, 2020. Ethical clearance was obtained from the Institutional Review Committee with reference no. 040320201 on Mar 4 2020.

A descriptive cross-sectional study was done in 200 students. Convenient sampling method was used and the sample size (n) was calculated with prevalence of 50% as follows:

$$\begin{array}{ll} \text{n} & ={\text{Z}}^{\text{2}}\times \text{p}\times \text{q}/{\text{e}}^{\text{2}}\\ & =1.96^{\text{2}}\times 0.5\times (1-0.5)/0.07^{\text{2}}\\ & =196 \end{array}$$

Where,

Z = 1.96 for 95% confidence interval
p = prevalence (50%)
q = 1-p
e = margin of error = 7%
n = minimum number of the sample size required

Two hundred undergraduate students studying in the first and second years of MBBS and BDS and willing to participate in the study were enrolled. Written consent was taken from the participants. A Performa was designed to verify associated risk factors like history of skin infection (organism associated, recurrence), recent hospital admission, OPD visit, recent antibiotic usage.

For microbiological confirmation of S. aureus colonization followed by the MRSA detection, the nasal swab was collected from anterior nares of the participants using sterile swab sticks and inoculated onto Mannitol salt agar. Yellow colonies yielded on Mannitol salt agar were preliminarily identified as S. aureus. Further confirmation was done using Gram's stain, Catalase, and Coagulase test using standard Microbiological techniques.

Antibiotic susceptibility testing of the S. aureus isolates was done using a modified Kirby-Bauer disc diffusion method.^5^ MRSA detection was done using cefoxitin (30μg) disc following the modified Kirby-Bauer disc diffusion technique. The S. aureus isolates exhibiting ≤21 mm of inhibition zone around the cefoxitin disk were confirmed as MRSA. Reference strains S. aureus ATCC 25923 and ATCC 43300 were used as negative and positive controls, respectively.^6^

The data obtained were computed and analyzed using SPSS 16.0 Version.

## RESULTS

A total of 200 participants were enrolled in the study. All the participants were in the age group of 18-25 years. Among the participants, 9 (4.5%) participants were found to be harboring MRSA.

Among the participants with MRSA colonization, associated risk factors were observed. However, 4 (44.44%) of the colonizers did not have a history of any associated risk factors (Table 1).

**Table 1:** Associated risk factors for MRSA colonization.

Associated risk factors	n (%)
Past skin infections	1 (11.11)
Hospital admission	0 (0)
Recent OPD visit	2 (22.22)
Recent antibiotic usage	2 (22.22)
No associated risk factors	4 (44.44)
Total	9 (100)

**Figure 1:**
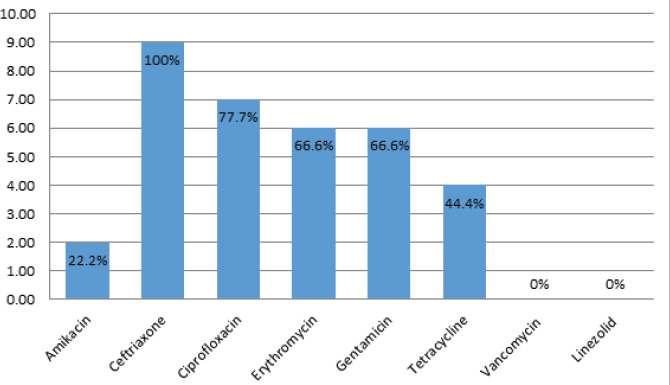
Antibiotic resistance pattern of MRSA. (n=9).

The Antimicrobial susceptibility testing of the MRSA isolates showed the highest antibiotic resistance prevalence against Ceftriaxone 9 (100%) (Figure 1).

## DISCUSSION

Nasal carriage of MRSA in colonized persons acts as a potential endogenous reservoir for clinical infections and a cross-colonization source for the spread in community.^7^ Medical students, though overlooked, could be a significant potential source of infection in healthcare settings.

In our study, a total of 200 participants studying in the first and second year of MBBS and BDS were enrolled for the study. All were in the age group of 18-25 years. Among the participants, 4.5% of participants were found to be harboring MRSA. Similar studies involving medical students from Nepal by Ansari et al. reported the prevalence of MRSA colonization to be 4%, while Bhatt et al. reported the prevalence to be 40%.^6,8^ Nasal carriage to MRSA is multifactorial and depends on various host factors.^9^ This might have been reflected in the variability in MRSA colonization in the studies mentioned above.

Recent antibiotic usage and contact with patients having SSTIs have been associated with MRSA colonization and infection.^1^ In our study, among the participants colonized with MRSA, 2 participants had a recent OPD visit, and 2 had a recent history of antibiotic usage. Purulent SSTIs are the most common clinical manifestations of CA-MRSA.^1^ This may explain the fact that 1 MRSA colonizer in our study had recurrent skin infections in the past. However, 44.45% did not have a history of any associated risk factors. The MRSA carriers were advised to apply mupirocin ointment in the anterior nares (2-3 times per day for 5 days) for decolonization.

The Antimicrobial susceptibility testing of MRSA isolates exhibited the highest level of drug resistance to ceftriaxone 100%, followed by ciprofloxacin 77.7%, gentamicin 66.6%, and erythromycin 66.6%, respectively. None of the isolates exhibited resistance to vancomycin and linezolid. This finding is in accordance with studies that were done in Nepal by Ansari et al., Raut et al.^6,10^

In Nepal, medical and dental students are introduced to clinical cases from the third year of their course. But the knowledge they have about healthcare-associated infections and screening methods, common infection control practices is inadequate, thus posing them as a potential source of infection in healthcare settings. This indicates that early on from the undergraduate course, medical and dental students should be introduced to the concepts of MRSA carriers, screening methods available, and hospital infection control measures.

The study's limitations were that intermittent carriers may have been missed as the study was cross-sectional, and only one method of screening MRSA was used. Moreover, detail follows up of the students regarding their knowledge regarding MRSA was not done. Molecular characterization of MRSA was not done due to resource constraints.

## CONCLUSIONS

The nasal carriage of MRSA by apparently healthy medical and dental students may inadvertently play a role in changing the epidemiology of MRSA in healthcare settings. Preventive measures and educating the students is needed to avoid outbreaks.
